# Endogenous endophthalmitis secondary to subclinical pyelonephritis

**DOI:** 10.1002/jhm.70244

**Published:** 2026-01-20

**Authors:** Riya N. Soni, Ariadna Perez‐Sanchez, Robert Nathanson

**Affiliations:** ^1^ Department of Medicine, Division of Infectious Diseases University of Texas Health San Antonio San Antonio Texas USA; ^2^ Hospital Medicine Section, Division of General Internal Medicine Spencer Fox Eccles School of Medicine at the University of Utah Salt Lake City Utah USA; ^3^ Division of Hospital Medicine University of Texas Health San Antonio San Antonio Texas USA; ^4^ Section of Hospital Medicine South Texas Veterans Health Care System San Antonio Texas USA

## CASE DESCRIPTION

A 45‐year‐old woman with poorly controlled diabetes mellitus presented with 2 days of vision loss in her left eye associated with eye pain, photophobia, and drainage. Conjunctiva was erythematous, edematous, and protruding, and extraocular movements were limited. Ophthalmological examination revealed 1.5 mm hypopyon and mobile vitreous debris. Vitreous cultures were collected, and vancomycin and ceftazidime were injected intravitreally. Intravenous vancomycin and ceftazidime were started, along with prednisone, moxifloxacin, and atropine eye drops for presumed endogenous endophthalmitis (EE). Blood and urine cultures returned positive with *Klebsiella pneumoniae*. Computerized tomography of the abdomen revealed bilateral pyelonephritis. She was diagnosed with EE secondary to *K. pneumoniae* bacteremia from bilateral pyelonephritis. Mechanical vitrectomy was performed. She improved with intravenous meropenem followed by oral levofloxacin upon discharge.

EE is a vision‐threatening infection of the intraocular cavity due to hematogenous spread of pathogens. Primary sources of EE include endocarditis (40%), urinary tract infections, intravenous catheters, and drug use. Most common pathogens in Western countries are *Staphylococcus aureus* (25%), Streptococci (30%–50%), and Gram‐negative bacilli (30%). *K. pneumoniae* is uncommon in the United States but a leading cause in Asia due to its association with liver abscesses.^1^ Diabetes mellitus is a major risk factor for EE due to *K. pneumoniae*.^2^ Our patient's poorly controlled diabetes mellitus (hemoglobin A1C of 12.2%) likely contributed to development of *K. pneumoniae* pyelonephritis with minimal symptoms, including lack of fever or leukocytosis. Most importantly, clinicians must promptly recognize EE to avoid delays in management and obtain ophthalmological consultation to minimize risk of vision loss.

## CONFLICT OF INTEREST STATEMENT

The authors declare no conflicts of interest.

## ETHICS STATEMENT

**Figure 1:**
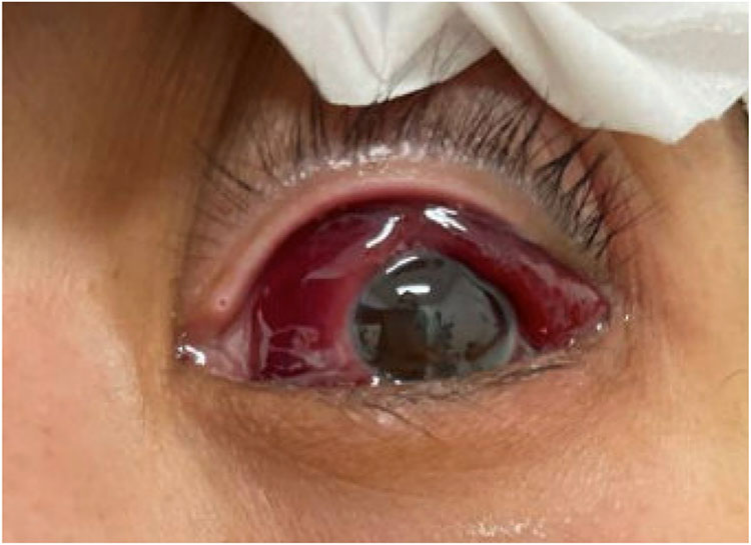
Left eye on admission. Conjunctiva was erythematous, edematous, and protruding, and ophthalmological exam revealed a 1.5 mm hypopyon.

**Figure 2:**
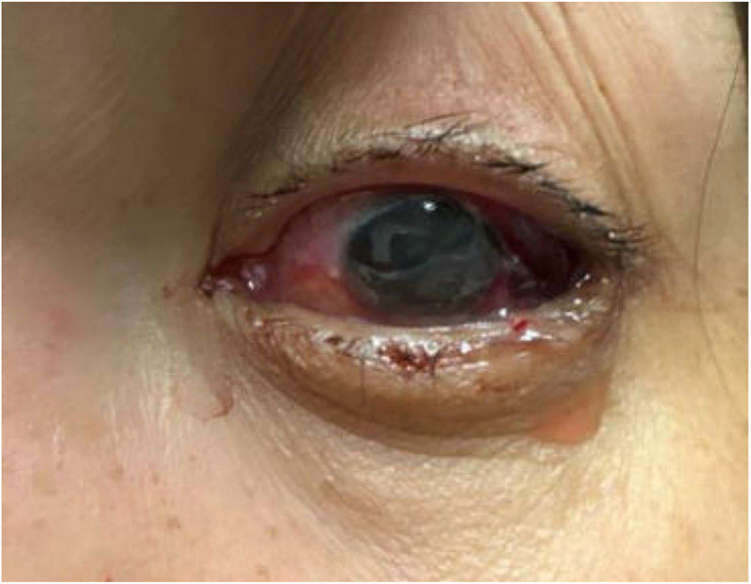
Left eye after treatment. Improvement in the conjunctival erythema and edema is seen within 24 h after treatment with intravitreal and systemic antibiotics.

This patient provided verbal consent to share this case among the medical team locally; however, we attempted to contact the patient multiple times using all provided phone numbers and contacts in her medical record, but we were unable to obtain written consent. Most importantly, the photos (Figures 1 and 2) are limited to the eye, are de‐identified, and cannot be traced to a specific patient.

## Data Availability

All patient data related to visual vignette is available upon request.
